# Adipose tissue expansion in obesity, health, and disease

**DOI:** 10.3389/fcell.2023.1188844

**Published:** 2023-04-26

**Authors:** Ursula White

**Affiliations:** Clinical Science Division, LSU Pennington Biomedical Research Center, Baton Rouge, LA, United States

**Keywords:** adipose tissue, adipose expansion, adipocyte, adipogenesis, obesity, human, metabolic health

## Abstract

White adipose tissue (WAT) expands under physiological conditions via an increase in adipocyte size (hypertrophy) and/or number (hyperplasia; adipogenesis), and the ability of WAT to expand to accommodate energy demands is a significant determinant of metabolic health status. Obesity is associated with impaired WAT expansion and remodeling, which results in the deposition of lipids to other non-adipose organs, leading to metabolic derangements. Although increased hyperplasia has been implicated as a cornerstone in promoting healthy WAT expansion, recent developments suggest that the role of adipogenesis as a contributing factor in the transition from impaired subcutaneous WAT expansion to impaired metabolic health remains up for debate. This mini-review will summarize recent developments and highlight emerging concepts on the features of WAT expansion and turnover, and the significance in obesity, health, and disease.

## 1 Introduction

White adipose tissue (WAT) is the primary site for energy storage in the form of triacylglycerols (TGs) and is an important regulator of whole-body energy homeostasis (Cypess, 2022). Due to its high plasticity, WAT retains the ability to expand, reduce, and remodel during adulthood to accommodate changes in energy balance in response to a variety of metabolic stimuli, including obesity, diet, and exercise. The patterns of WAT expansion vary amongst the population and during metabolic perturbations, and these mechanisms continue to be uncovered. WAT mass is regulated by dynamic changes in adipocyte volume, via TG synthesis and breakdown, as well as adipocyte formation (i.e., hyperplasia; adipogenesis) and death. It is now appreciated that there is constant turnover (synthesis/formation and removal/death) of WAT TGs (Strawford et al., 2004; Arner et al., 2011) and adipocytes (Strawford et al., 2004; Spalding et al., 2008), respectively; and it is estimated that ∼8% of adipocytes are replaced each year in adults (Spalding et al., 2008).

WAT expansion under physiological conditions involves via both hypertrophy (enlargement of existing adipocytes’ size) and hyperplasia (increase in adipocyte number; adipogenesis), and evidence suggests that the capacity of subcutaneous WAT to expand is a key determinant of obesity-related metabolic dysregulation (Danforth, 2000; Sethi and Vidal-Puig, 2007). The adipose tissue expandability hypothesis postulates that impaired subcutaneous WAT expansion in response to energy demands can lead to ectopic lipid deposition in non-adipose organs and contribute to the development of insulin resistance and type 2 diabetes (Virtue and Vidal-Puig, 2010). Arner et al. reported that greater hypertrophy was linked to lower adipocyte number and insulin resistance, independent of sex and adiposity, while greater hyperplastic expansion was associated with better insulin sensitivity (Arner et al., 2010). Additional studies also suggest that impaired adipogenesis, or restricted hyperplasia, is linked to metabolic dysfunction (Weyer et al., 2000; Lessard et al., 2014), while a higher population of small adipocytes (e.g., adipogenesis) is associated with better glycemic and lipid profiles (Arner et al., 2010; Hoffstedt et al., 2010). However, the role of impaired adipogenesis as a contributing factor in the pathogenesis of obesity-related disorders remains debatable (White and Ravussin, 2019). Other studies reported that individuals with insulin resistance or T2DM have a higher proportion of small adipocytes, suggesting hyperplastic expansion, as compared to healthy individuals (McLaughlin et al., 2007; Pasarica et al., 2009; McLaughlin et al., 2014), and these observations imply that impaired expansion of small adipocytes is associated with insulin resistance (McLaughlin et al., 2007; McLaughlin et al., 2014; White et al., 2022).

Despite the significant role of WAT expansion in human health and pathology, the mechanistic underpinnings, notably the kinetics of adipose tissue components (adipocytes and TGs), are not fully understood. This is due, in part, to the slow turnover rate of WAT components. This mini review will discuss the dynamics of WAT expansion in humans during obesity, changes in energy balance (e.g., weight gain and loss), and exercise as relates to metabolic health and disease in humans, as well as some emerging, state-of-the-art methodologies to assess the features of WAT expansion.

## 2 Challenges and novel approaches to measure adipose tissue expansion

The expansion and turnover of WAT (lipids and cells) have been difficult to study given the lack of appropriate methods; thus, the underlying mechanisms are not fully understood. Indirect measures, such as fat cell size and molecular markers of cell proliferation and death, are informative, but do not provide an integrative evaluation of turnover. Furthermore, the unspecific labeling of nucleotides with ^3^H-thymidine or BrdU, as well as the toxicity of the label, make these approaches inapplicable to humans. A more recent *in vitro* approach is the use of three-dimensional (3D) adipose tissue culture models, which can utilize human WAT stem cells that differentiate and organize into adipose organ-like structures or adipose explants in culture, thus, providing a more physiological setting to investigate adipocyte function (Murphy et al., 2019). Nevertheless, *in vivo* approaches are necessary to capture the dynamic changes that occur during the various facets of WAT expansion and turnover within the natural environment of the adipose tissue.

Spalding et al. (2008) introduced an *in vivo* method to study fat cell and lipid turnover in humans by measuring the incorporation of atmospheric ^14^C, derived from above ground nuclear bomb tests, into the adipocytes (Arner et al., 2011). The Hellerstein group developed another innovative method (Busch et al., 2007) using the incorporation of the stable isotope deuterium (^2^H) into adipose cells and lipids that has been validated to provide physiological, quantitative measures of WAT turnover *in vivo* (Neese et al., 2002; Strawford et al., 2004). While the ^14^C-labeling method has provided informative retrospective assessments of WAT expansion and turnover, the ^2^H-labeling approach can measure dynamic changes in WAT during prospective intervention studies (White and Ravussin, 2019). Studies using metabolic labeling with stable isotopes can fill a substantial knowledge gap surrounding the dynamics of WAT remodeling and plasticity in humans.

## 3 Adipose tissue expansion in obesity

Obesity, characterized by excessive adiposity, can lead to dysregulation of WAT expansion and function, resulting in lipotoxicity and obesity-related comorbidities (Virtue and Vidal-Puig, 2010). Because *in vitro* data provide evidence to support the adipose tissue expandability hypothesis, one group investigated the relationship between the estimated manner of WAT expansion and the *in vivo* generation of adipocytes, as assessed by the ^14^C-labeling approach, in both lean subjects and individuals with obesity (Arner et al., 2010). Individuals displaying more hypertrophic fat expansion produced fewer adipocytes *in vivo* per year than individuals displaying more hyperplastic expansion. Another study assessed *in vivo* kinetics in the subcutaneous abdominal and femoral WAT depots of women with obesity using the ^2^H-labeling protocol and reported that higher *in vivo* adipocyte formation rates were positively correlated with increased adiposity [body mass index (BMI) and % body fat] (White et al., 2016). Interestingly, higher (not lower) *in vivo* adipogenesis was positively associated with visceral adipose tissue content and negatively associated with insulin sensitivity (White et al., 2017). These data challenge the adipose tissue expandability hypothesis and provide the first evidence of an association between facets of impaired metabolic health and higher, not lower, *in vivo* adipose cell formation.

TGs are a metabolically active pool and estimated to be replaced (i.e., turnover) ∼6 times during the ∼10 years lifespan of a healthy adipocyte. However, obesity, which is associated with impaired lipid metabolism, is characterized by decreased adipocyte TG turnover, as TGs are estimated to be replaced only ∼3 times during the lifespan an adipocyte in individuals with obesity (Arner et al., 2011). This implies that high TG storage coupled with low removal (via lipolysis), or low TG turnover, may be important determinants of obesity (Arner et al., 2011). Other observations using the ^2^H-labeling approach reported that Black women had lower TG synthesis rates as compared to White women with obesity (White et al., 2018), who were also shown to have enhanced insulin sensitivity vs. Black women (DeLany et al., 2014). Another group reported that TG synthesis rates were significantly higher in insulin-sensitive vs. insulin-resistant individuals (Allister et al., 2015). Overall, these data suggest that higher adipose TG turnover is associated with favorable metabolic outcomes. Notably, a recently published study (Arner et al., 2018) implicated lipolysis as an important predictor of future weight gain and impaired glucose metabolism.

## 4 Adipose tissue expansion during changes in energy balance

To date, there have been no experimental overfeeding studies to examine the dynamics of WAT expandability *in vivo* during weight gain. Two studies (Johannsen et al., 2014; McLaughlin et al., 2016) have reported that individuals with smaller mean adipocyte size at baseline had the most impaired metabolic health outcomes (e.g., greater decline in insulin sensitivity) in response to overfeeding and weight gain, as compared to those with a larger adipocyte size. In addition, one group recently reported that an increased proportion of small adipocytes (i.e., hyperplasia) in subcutaneous WAT is associated with impaired (not improved) metabolic health outcomes, specifically visceral and ectopic fat accumulation in the liver, during weight gain in response to overfeeding (White et al., 2022). These findings imply the presence of small adipocytes with a decreased capacity to accommodate lipid. Interestingly, although few studies have assessed depot differences in WAT expansion in humans, one study suggested depot-specific fat expansion in response to overfeeding, with mainly hypertrophy in the subcutaneous abdominal WAT and primarily hyperplasia in the subcutaneous femoral (Tchoukalova et al., 2010). Additional *in vivo* assessments during overfeeding interventions are necessary to better characterize the influence of WAT expansion during positive energy balance in the pathogenesis of metabolic disorders.

There is a paucity of data on the dynamics of WAT plasticity and remodelling during weight loss. Interestingly, one investigation reported that adipose cell formation in the subcutaneous WAT was negatively associated with the change in body weight during the ^2^H-labeling period, suggesting that women with greater weight loss had higher *in vivo* adipogenesis (White et al., 2017). Further investigations are necessary to better understand WAT expansion and remodelling during weight loss (i.e., caloric restriction, bariatric surgery, etc.) and determine how these adaptations could influence weight regain and weight cycling.

## 5 Adipose tissue expansion and exercise

The favorable effects of exercise on cardiovascular health and skeletal muscle are well-established, but rodent studies suggest that exercise-induced adaptations in the WAT may also influence overall metabolic health (Stanford et al., 2015). Although some important WAT adaptations in response to exercise have been reported, including changes in morphology and decreased adipocyte size (Despres et al., 1984; Mauriege et al., 1997), no studies have reported the effects of exercise on WAT turnover *in vivo* in humans. One study using the ^2^H-labeling protocol demonstrated that exercise (4 weeks of voluntary wheel running) significantly reduced new adipocyte formation (e.g., adipogenesis) in the WAT of both male and female mice *in vivo* (Allerton et al., 2021). Despite the very limited data available, these results suggest that exercise induces WAT remodeling in such a manner that reduces the need for new adipocyte formation, possibly due to enhanced metabolic efficiency and lifespan of existing adipocytes. Of note, higher adipogenesis could indicate a need for new adipocyte formation, due to existing adipocytes’ fragility and death, which can lead to the recruitment of macrophages, unfavorable remodeling and inflammation (Strissel et al., 2007). Hence, lower hyperplasia could be a critical and metabolically favorable exercise-mediated WAT adaptation. Human studies to assess the effects of exercise on the dynamics of WAT expansion *in vivo* have yet to be reported.

## 6 Discussion

Given that the capacity for WAT expansion is a significant determinant of obesity-related complications, further investigations are necessary to better elucidate the important facets of WAT expandability and turnover in humans. Of note, more recent studies have identified the presence of white adipocyte subpopulations in WAT with distinct functions (Bilson et al., 2023), suggesting that adipocytes are not a uniform cell type. This presents new challenges to better understand WAT biology, as changes in the presence, function, and/or turnover of adipocyte subpopulations can contribute to obesity-related metabolic diseases.

Isotopic labeling methodologies to assess *in vivo* lipid and cell kinetics are a substantive departure from indirect and *in vitro* methods; and new insights derived from these emerging, cutting-edge approaches will advance our understanding of the important changes in WAT function that occur during changes in energy balance and in conditions of obesity and disease. Notably, the ^2^H-labeling method can provide comprehensive, quantitative measures of WAT turnover during a variety of prospective intervention studies, such as diet, exercise, and pharmacological treatments. This knowledge can facilitate the future development of therapeutic targets and treatments for obesity and related disorders that are focused on WAT.
